# Artificial intelligence literacy and innovative behavior among clinical nurses: the roles of AI self-efficacy and organizational innovation climate

**DOI:** 10.3389/fpubh.2026.1958854

**Published:** 2026-10-22

**Authors:** Jiahang Li, Xianyong Liu, Yang Cao, Caiyan Tang, Jianwei Zhou

**Affiliations:** 1 Medical Department of Hunan Normal University, Changsha, Hunan, China; 2 Nursing Department of Yueyang Hospital Affiliated with Hunan Normal University (Yueyang People's Hospital), Yueyang, Hunan, China

**Keywords:** clinical nurses, artificial intelligence literacy, artificial intelligence self-efficacy, nurse innovative behavior, organizational innovation climate

## Abstract

**Objective:**

To investigate the current status and related factors of innovative behavior among clinical nurses in tertiary hospitals and explore the relationships and pathways among artificial intelligence literacy, AI self-efficacy, organizational innovation climate, and nurse innovative behavior, for providing a basis for targeted nursing management strategies.

**Methods:**

This cross-sectional study was conducted on 850 nurses from six tertiary medical institutions using a stratified survey approach in Yueyang city, Hunan province, from June to July 2026, in the General Information Questionnaire, Artificial Intelligence Literacy Scale, Artificial Intelligence Self-Efficacy Scale, Organizational Innovation Climate Scale, and Nurse Innovative Behavior Scale were used. AMOS 24.0 was used to conduct confirmatory factor analysis to assess the model fit of the scales. SPSS 26.0 was used for descriptive statistics, univariate analysis, Pearson correlation analysis, and multiple linear regression. PROCESS macro version 4.2 Models 4 and 14 tested mediation and moderated mediation effects.

**Results:**

A total of 850 valid questionnaires were obtained, with a valid questionnaire rate of 95.3%. The mean nurse innovative behavior score was 37.93±6.50. The measurement model for the four scales showed good fit, and the scales demonstrated good convergent validity and some evidence of discriminant validity. Artificial intelligence literacy, artificial intelligence self-efficacy, organizational innovation climate, and innovative behavior showed positive associations with one another (r = 0.609–0.797, *P* < 0.01). Educational level, professional title, participation in research or innovation activities, artificial intelligence literacy, artificial intelligence self-efficacy, and organizational innovation climate were factors associated with nurse innovative behavior and explained 60.9% of the total variance. Artificial intelligence self-efficacy partially mediated the relationship between artificial intelligence literacy and nurse innovative behavior. The indirect effect was 0.322, with a 95% CI of 0.243–0.403, accounting for 52.90% of the total effect. Organizational innovation climate moderated the relationship between artificial intelligence self-efficacy and nurse innovative behavior (b = 0.051, *P* < 0.01). The index of moderated mediation was 0.041, with a 95% CI of 0.009–0.078.

**Conclusion:**

AI literacy was closely related to nurse innovative behavior, with AI self-efficacy as a partial mediator and organizational innovation climate as a moderator. Nursing management may focus on AI competencies, application confidence, and organizational support.

## 1 Introduction

Nurses' innovative behavior (1) refers to the behavioral process through which nurses actively generate, promote, and implement new methods or solutions based on clinical nursing problems and patient needs. This behavior helps optimize nursing processes, improve the quality and efficiency of nursing services, and improve patient outcomes (2). Previous studies have shown that the overall level of innovative behavior among clinical nurses is moderate and varies across different groups of nurses (3). Compared with the generation of innovative ideas, nurses show relatively lower performance in obtaining support and promoting implementation (4). This indicates that there are still barriers to moving innovation from ideas into practice. Identifying factors related to nurses' innovative behavior is important for promoting nursing innovation and improving the quality of nursing services. Existing studies have mainly focused on factors such as professional competence, psychological resources, and organizational environment (5, 6). Most of these studies have examined a single factor or the relationships between variables. As artificial intelligence is gradually integrated into nursing practice, the ways in which nurses identify problems, obtain information, and develop improvement plans are changing. Existing research perspectives may not fully capture the new changes in nurses' innovative behavior in the context of artificial intelligence. In 2025, the National Health Commission and other departments issued the Implementation Opinions on Promoting and Regulating the Development of “Artificial Intelligence Plus Healthcare” Applications (7), which proposed expanding the application scenarios of artificial intelligence in healthcare. Artificial intelligence provides new technological support for optimizing nursing services. At the same time, it places higher requirements on nurses' understanding of technology, assessment of appropriate application scenarios, and standardized use of technology (8). Therefore, it is necessary to further identify factors associated with nurses' innovative behavior in the context of artificial intelligence.

The Ability–Motivation–Opportunity (AMO) theory proposes that individual behavior is jointly shaped by ability, motivation, and opportunity (9, 10). Ability refers to the knowledge and skills required to perform a task. Artificial intelligence literacy refers to an individual's comprehensive ability to understand, use, and evaluate artificial intelligence technologies (11) and reflects the ability base for nurses to apply artificial intelligence in clinical practice. Previous studies have found that artificial intelligence literacy is positively correlated with innovative behavior (12). However, nurses' innovative behavior is not related only to their ability to use artificial intelligence. Artificial intelligence self-efficacy refers to an individual's confidence in their ability to learn, use, and adapt to artificial intelligence technologies (13). Within AMO theory, motivation emphasizes an individual's willingness to take action and sustain effort. Social cognitive theory also suggests that self-efficacy is related to behavioral choices, effort, and persistence. Accordingly, artificial intelligence self-efficacy reflects a motivation-related psychological belief concerning nurses' willingness to put their artificial intelligence-related abilities into practice. Previous studies have shown that artificial intelligence literacy is positively correlated with artificial intelligence self-efficacy (14, 15). Artificial intelligence self-efficacy is also closely related to nurses' innovative behavior (16). In addition, AMO theory emphasizes the importance of opportunity factors for individual behavior. Opportunity refers to the external resources and support available to individuals when engaging in practice. Organizational innovation climate refers to nurses' overall perceptions of the extent to which their hospital or department encourages innovation, provides resources, and supports innovative practice (17), and reflects the opportunities and support provided by the organization for nurses to engage in innovative practice. Previous studies have shown that organizational innovation climate is positively correlated with nurses' innovative behavior (18) and may be related to the strength of the association between self-efficacy and innovative behavior (19). Accordingly, the positive association between artificial intelligence self-efficacy and nurse innovative behavior may be stronger at higher levels of organizational innovation climate.

Existing studies have mainly examined the relationship between each variable and nurses' innovative behavior separately. Recent studies have also applied AMO theory to examine the relationships between factors such as organizational innovation climate and nurses' innovative behavior (20). However, in the context of artificial intelligence in nursing, research remains limited that integrates individual ability, motivation-related beliefs, and organizational opportunity conditions within a single theoretical framework and comprehensively examines their relationships with nurse innovative behavior and the potential patterns underlying these associations. Therefore, guided by AMO theory and incorporating the social cognitive perspective on self-efficacy, this study investigated the current status and related factors of innovative behavior among clinical nurses. A moderated mediation model was further constructed and tested to provide a more comprehensive examination of these relationships. It also explored the relationships among artificial intelligence literacy, artificial intelligence self-efficacy, organizational innovation climate, and innovative behavior. The findings may provide a basis for developing nursing management measures to promote innovative behavior among clinical nurses.

## 2 Research hypotheses

According to AMO theory, artificial intelligence literacy reflects the ability basis for nurses to apply artificial intelligence, whereas artificial intelligence self-efficacy reflects a motivation-related belief in putting these abilities into practice. Social cognitive theory also suggests that self-efficacy is closely associated with individuals' behavioral choices and sustained engagement. Previous studies have shown that artificial intelligence literacy is positively correlated with artificial intelligence self-efficacy (14, 15), and that artificial intelligence self-efficacy is also associated with nurses' innovative behavior (16). Based on this evidence, Hypothesis 1 was proposed.

AMO theory also emphasizes the importance of external opportunity conditions. Organizational innovation climate reflects organizational support and resources for innovation and may be associated with the strength of the association between artificial intelligence self-efficacy and innovative behavior. Based on this reasoning, Hypothesis 2 was proposed. The hypothesized model is shown in Figure 1.

**Figure 1:**
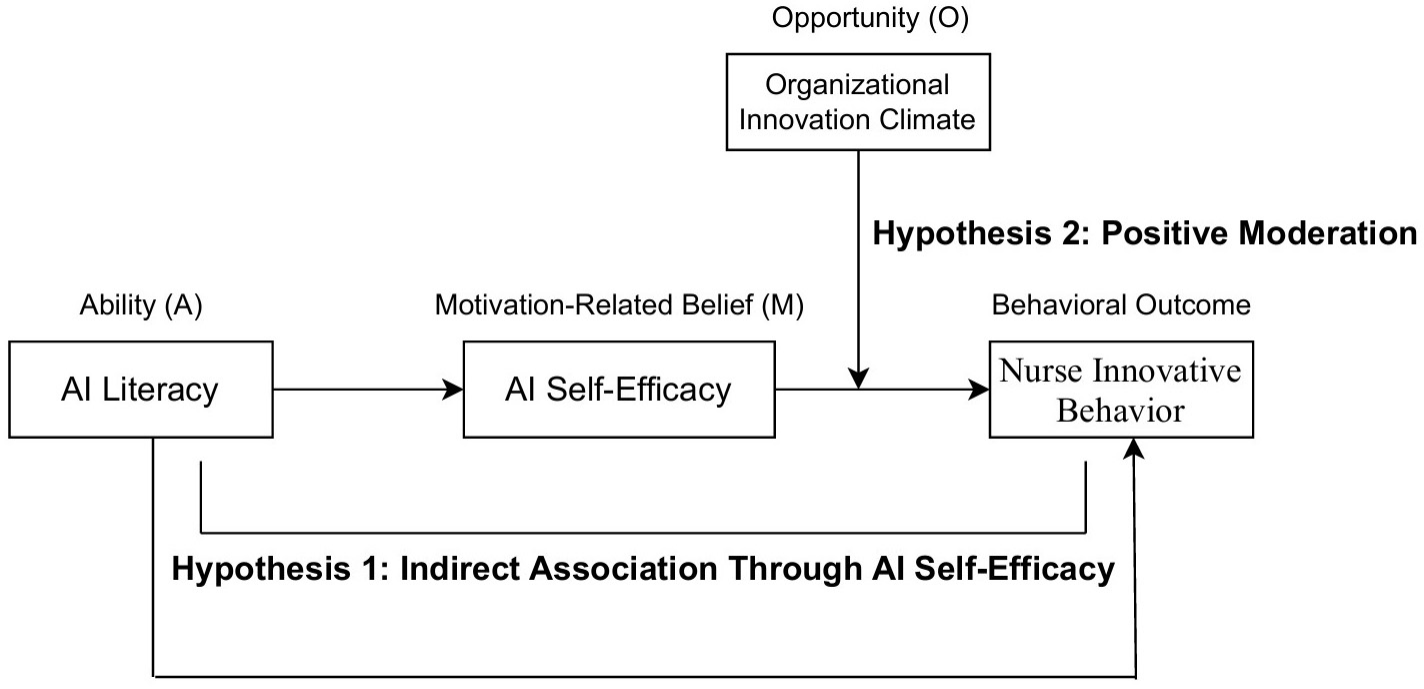
The hypothesized moderated mediation model.

**Hypothesis 1:** Artificial intelligence self-efficacy mediates the relationship between artificial intelligence literacy and innovative behavior.

**Hypothesis 2:** Organizational innovation climate positively moderates the relationship between artificial intelligence self-efficacy and innovative behavior. Specifically, the higher the level of organizational innovation climate, the stronger the positive association between artificial intelligence self-efficacy and innovative behavior.

## 3 Methods

### 3.1 Study design and participants

This study used a multicenter cross-sectional design. A stratified survey was conducted among clinical nurses from six tertiary medical institutions in Yueyang City, Hunan Province, from June to July 2026. The six institutions included two tertiary hospitals and four Grade-A tertiary hospitals, of which four were general hospitals and two were specialized hospitals, thereby covering different hospital grades and types. Within each hospital, nursing units were stratified into three categories: internal medicine, surgery, and other nursing units, with separate recruitment quotas established for each stratum to increase the diversity of the sample in terms of clinical work settings and organizational environments. The study was reported in accordance with the reporting guidelines for observational studies.

The inclusion criteria were as follows: (1) holding a Chinese nurse practice qualification and having completed professional registration; (2) currently engaging in clinical nursing work at one of the selected medical institutions, with at least 1 year of clinical nursing experience; and (3) understanding the purpose of the study and voluntarily agreeing to participate. The exclusion criterion was being absent from work during the survey because of sick leave, maternity leave, continuing education, or other reasons.

### 3.2 Sample size

During the study design stage, both the multicenter survey structure and the sample distribution across strata were considered. At least 120 nurses were planned to be recruited from each hospital, including at least 40 from internal medicine, 40 from surgery, and 40 from other nursing units, resulting in a target sample size of 720 participants. To further assess whether the sample size was adequate for the primary analytical model, a power analysis was conducted using G^*^Power 3.1.9.7 (21), based on the increase in R^2^ attributable to the interaction term in the outcome equation of PROCESS Model 14. With an effect size of f^2^ = 0.02, α = 0.05, statistical power (1–β) = 0.95, one tested predictor, and four total predictors in the outcome equation, the estimated minimum required sample size was 652. A total of 850 nurses were ultimately included in the study.

### 3.3 Study instruments

#### 3.3.1 General information questionnaire

The questionnaire was developed by the researchers according to the study objectives and relevant literature. It included 14 variables: gender, age, educational level, type of hospital, years of work experience, professional title, marital status, department, work content, employment type, participation in artificial intelligence-related training, frequency of artificial intelligence tool use, deployment of artificial intelligence systems in the department or hospital, and participation in nursing research or innovation improvement activities during the previous 3 years. Artificial intelligence-related training referred to whether participants had attended courses, lectures, or other training on artificial intelligence knowledge or applications organized by hospitals, schools, or other institutions. The frequency of artificial intelligence tool use was categorized according to the number of times such tools were used in daily practice. Deployment of artificial intelligence systems referred to whether artificial intelligence-assisted systems had been implemented in actual practice in the participant's department or hospital at the time of the survey.

#### 3.3.2 Artificial intelligence literacy scale

The Artificial Intelligence Literacy Scale was developed by Wang et al. (22) in 2023. It includes four dimensions: awareness, use, evaluation, and ethics. Each dimension contains three items, with a total of 12 items. The scale uses a 7-point Likert scoring system, ranging from 1 for “strongly disagree” to 7 for “strongly agree.” The total score ranges from 12 to 84. A higher score indicates a higher level of artificial intelligence literacy. The Cronbach's α coefficient of the original scale was 0.83, and the Cronbach's α coefficient in the present study was 0.90.

#### 3.3.3 Artificial intelligence self-efficacy scale

The Artificial Intelligence Self-Efficacy Scale was developed by Wang et al. (23) in 2024. It includes four dimensions: assistance (7 items), anthropomorphic interaction (5 items), comfort with artificial intelligence (6 items), and technical skills (4 items), with a total of 22 items. The scale uses a 7-point Likert scoring system, ranging from 1 for “strongly disagree” to 7 for “strongly agree.” The total score ranges from 22 to 154. A higher score indicates a higher level of artificial intelligence self-efficacy. The Cronbach's α coefficient of the original scale was 0.98, and the Cronbach's α coefficient in the present study was 0.96.

#### 3.3.4 Organizational innovation climate scale

The Organizational Innovation Climate Scale was developed by Qian et al. (24) in 2016. It includes three dimensions: organizational innovation incentives (7 items), resource provision (6 items), and management practices (8 items), with a total of 21 items. The scale uses a 5-point Likert scoring system, ranging from 1 for “strongly disagree” to 5 for “strongly agree.” The total score ranges from 21 to 105. A higher score indicates a more positive organizational innovation climate perceived by nurses. The Cronbach's α coefficient of the original scale was 0.94, and the Cronbach's α coefficient in the present study was 0.92.

#### 3.3.5 Nurse innovative behavior scale

The Nurse Innovative Behavior Scale was developed by Bao et al. (25) in 2012. It includes three dimensions: generating ideas (3 items), obtaining support (4 items), and realizing ideas (3 items), with a total of 10 items. The scale uses a 5-point Likert scoring system, ranging from 1 for “never” to 5 for “very frequently.” The total score ranges from 10 to 50. A higher score indicates a higher level of nurse innovative behavior. The Cronbach's α coefficient of the original scale was 0.88, and the Cronbach's α coefficient in the present study was 0.87.

### 3.4 Data collection

An online survey was conducted through the Wenjuanxing platform. After permission was obtained from the nursing management departments of the six hospitals, the nursing departments of each hospital assisted the head nurses in forwarding the questionnaire link or QR code to their departments. The head nurses were responsible only for distributing the questionnaire and did not access individual responses or link participation in the survey to work performance evaluations or other employment-related matters. The person responsible for the study at each hospital monitored recruitment progress based on the number of questionnaire submissions reported by the nursing units. Once the prespecified target of 120 questionnaires was reached, the head nurses were informed to stop inviting additional nurses to participate. The first page of the questionnaire described the study purpose, completion requirements, principles of anonymity and voluntary participation, and the use of the collected data. Participants could withdraw at any time before submitting the questionnaire. Each IP address or account was allowed to submit the questionnaire only once, and all items were mandatory.

Before the formal survey, a pilot study was conducted among 20 clinical nurses who met the inclusion criteria. The pilot study evaluated the comprehensibility of the questionnaire items and recorded the completion time. Data from the pilot study were not included in the final analysis. The results showed that all items were clearly expressed and required no revision. The questionnaire took approximately 5–10 min to complete. Based on this completion time, a completion time of < 5 min was used as one of the questionnaire quality-screening criteria. The quality-screening criteria included: (1) a completion time of < 5 min; (2) objective logical inconsistencies in demographic information, such as an estimated age at entry into nursing practice of clearly < 18 years based on age and years of work experience; and (3) selection of the same response option for all 65 scale items. A total of 892 questionnaires were collected. After excluding 42 questionnaires that did not meet the quality criteria, 850 valid questionnaires were included, resulting in a valid questionnaire rate of 95.3%.

### 3.5 Ethical considerations

This study was approved by the Ethics Committee of the Medical Department of Hunan Normal University (approval number: 2026-055). All participants provided informed consent and voluntarily participated in the study. The questionnaire data contained no names, contact information, specific hospital names, or other information that could directly identify individuals or institutions, and all data were used only for this study.

### 3.6 Statistical analysis

SPSS 26.0 was used for descriptive statistical analysis. Independent-samples *t* tests or one-way analysis of variance were used to compare nurse innovative behavior scores among participants with different general characteristics. Harman's single-factor test was used as a preliminary assessment of common method bias. Pearson correlation analysis was used to examine correlations among the variables. Multiple linear regression analysis was used to examine factors associated with nurse innovative behavior, and variance inflation factors (VIFs) were used to assess multicollinearity. Only variables that were statistically significant were reported in the regression results table. AMOS 24.0 was used to conduct confirmatory factor analysis to evaluate the model fit, convergent validity, and discriminant validity of the four main scales. PROCESS macro version 4.2 was used to test mediation and moderated mediation effects. Model 4 was used to examine the mediating role of artificial intelligence self-efficacy in the association between artificial intelligence literacy and nurse innovative behavior, with X, M, and Y representing the mean-centered total scores of artificial intelligence literacy, artificial intelligence self-efficacy, and nurse innovative behavior, respectively. In Model 14, organizational innovation climate was specified as the moderator W, and the M × W interaction was tested. Conditional indirect effects and the index of moderated mediation were also estimated. Models 4 and 14 were analyzed according to the prespecified theoretical model, without additional adjustment for demographic covariates. The significance of the effects was tested using 5,000 bootstrap resamples. A *P*-value of < 0.05 was considered statistically significant.

## 4 Results

### 4.1 General characteristics of clinical nurses and univariate analysis of nurse innovative behavior

A total of 892 questionnaires were collected in this study. After excluding 42 invalid questionnaires, 850 valid questionnaires were obtained, with a valid questionnaire rate of 95.3%. The total score of nurse innovative behavior was 37.93 ± 6.50. The mean age of the participants was 33.37 ± 5.96 years. Among them, 673 nurses (79.2%) had a bachelor's degree, 601 (70.7%) worked in general hospitals, 417 (49.1%) were supervisor nurses, and 707 (83.2%) were mainly engaged in clinical nursing. The remaining general characteristics are shown in Table 1.

**Table 1:** General characteristics of clinical nurses and univariate analysis of nurse innovative behavior.

Variable	Category	*n* (%)	t/F	*P*
Gender	Male	91 (10.7%)	0.361	0.719
	Female	759 (89.3%)		
Age (years)	20~30	378 (44.5%)	11.245	< 0.01
	31~40	390 (45.9%)		
	≥41	82 (9.6%)		
Educational level	Associate degree or below	163 (19.2%)	5.489	< 0.01
	Bachelor's degree	673 (79.2%)		
	Master's degree or above	14 (1.6%)		
Hospital type	General hospital	601 (70.7%)	0.368	0.713
	Specialized hospital	249 (29.3%)		
Years of work experience	1~5	150 (17.6%)	4.054	< 0.01
	6~10	231 (27.2%)		
	11~15	277 (32.6%)		
	≥16	192 (22.6%)		
Professional title	Nurse	69 (8.1%)	12.575	< 0.01
	Senior nurse	241 (28.4%)		
	Supervisor nurse	417 (49.1%)		
	Associate chief nurse or above	123 (14.5%)		
Marital status	Unmarried	202 (23.8%)	4.363	< 0.05
	Married	643 (75.6%)		
	Other	5 (0.6%)		
Department	Internal medicine	277 (32.6%)	0.735	0.480
	Surgery	263 (30.9%)		
	Other	310 (36.5%)		
Work content	Mainly clinical nursing	707 (83.2%)	−2.833	< 0.01
	Clinical nursing combined with teaching, management, research, other responsibilities	143 (16.8%)		
Employment type	Permanent staff	91 (10.7%)	0.221	0.802
	Contract staff	721 (84.8%)		
	Labor-dispatch staff	38 (4.5%)		
Participation in artificial intelligence-related training	Yes	280 (32.9%)	3.316	< 0.01
	No	570 (67.1%)		
Frequency of AI tool use	Never	84 (9.9%)	3.539	< 0.05
	≤ 1 time per week	144 (16.9%)		
	1~3 times per week	420 (49.4%)		
	Daily	202 (23.8%)		
Deployment of AI systems in the hospital	Yes	362 (42.6%)	−0.186	0.853
	No	488 (57.4%)		
Participation in nursing research or innovation improvement activities during the previous 3 years	Did not participate	504 (59.3%)	10.213	< 0.01
	Participated	297 (34.9%)		
	Led or was primarily responsible	49 (5.8%)		

Univariate analysis showed that nurse innovative behavior scores differed significantly according to age, educational level, years of work experience, professional title, marital status, work content, artificial intelligence training experience, frequency of artificial intelligence tool use, and participation in nursing research or innovation improvement activities during the previous 3 years (all *P* < 0.05). Details are presented in Table 1.

### 4.2 Common method bias test and confirmatory factor analysis

Harman's single-factor test was used to assess common method bias (26). The results showed that the first factor explained 38.601% of the total variance, which was below the critical threshold of 50%. This indicated that there was no serious common method bias in this study.

The measurement model of the four scales showed good fit: X^2^/df = 4.324 (< 5), CFI = 0.978 and TLI = 0.971 (both >0.90), and RMSEA = 0.063 and SRMR = 0.028 (both < 0.08). The standardized factor loadings of all dimensions ranged from 0.785 to 0.934, all exceeding 0.50. The composite reliability (CR) values of the four latent variables ranged from 0.860 to 0.933 (>0.70), and the average variance extracted (AVE) values ranged from 0.672 to 0.795 (>0.50), indicating good composite reliability and convergent validity. Correlations among the latent variables ranged from 0.668 to 0.853, all below 0.90, providing some evidence of discriminant validity.

### 4.3 Correlations among artificial intelligence literacy, artificial intelligence self-efficacy, organizational innovation climate, and innovative behavior

Pearson correlation analysis showed that artificial intelligence literacy, artificial intelligence self-efficacy, organizational innovation climate, and innovative behavior were significantly and positively correlated with one another. The correlation coefficients ranged from 0.609 to 0.797, with all *P* < 0.01. Details are shown in Table 2.

**Table 2:** Correlations among artificial intelligence literacy, artificial intelligence self-efficacy, organizational innovation climate, and nurse innovative behavior.

Variables	AI literacy	AI self-efficacy	Organizational innovation climate	Nurse innovative behavior
AI literacy	1			
AI self-efficacy	0.797^**^	1		
Organizational innovation climate	0.619^**^	0.631^**^	1	
Nurse innovative behavior	0.609^**^	0.633^**^	0.726^**^	1

^**^P < 0.01.

### 4.4 Multiple linear regression analysis of innovative behavior among clinical nurses

Nurse innovative behavior was treated as the dependent variable. General information variables that showed statistically significant differences in the univariate analysis as well as artificial intelligence literacy, artificial intelligence self-efficacy, and organizational innovation climate were entered into the multiple linear regression model. The results showed that educational level, professional title, participation in research or innovation activities, artificial intelligence literacy, artificial intelligence self-efficacy, and organizational innovation climate were significantly associated with nurse innovative behavior (R^2^ = 0.609, R^2^adj = 0.602, F = 86.546, *P* < 0.01). Collinearity diagnostics showed that the tolerance values ranged from 0.259 to 0.895 and the VIF values ranged from 1.118 to 3.855, indicating no serious multicollinearity. Variables that were statistically significant in the multiple linear regression analysis are presented in Table 3.

**Table 3:** Multiple linear regression analysis of factors associated with nurse innovative behavior among clinical nurses.

Independent variable		B	SE	β	t	*p*
Constant		−1.316	1.476	—	−0.891	0.373
Professional title	Supervisor nurse vs. Nurse	1.65	0.552	0.127	2.987	< 0.01
	Associate chief nurse or above vs. Nurse	1.777	0.680	0.096	2.613	< 0.01
Educational level	Bachelor's degree vs. Associate degree or below	1.036	0.373	0.065	2.78	< 0.01
	Master's degree or above vs. Associate degree or below	2.672	1.177	0.052	2.27	< 0.05
Participation in research or innovation activities	Participated vs. Did not participate	0.638	0.319	0.047	2.002	< 0.05
	Led or played a major role vs. Did not participate	1.362	0.659	0.049	2.066	< 0.05
AI literacy		0.076	0.023	0.124	3.348	< 0.01
AI self-efficacy		0.063	0.011	0.211	5.574	< 0.01
Organizational innovation climate		0.286	0.017	0.502	17.153	< 0.01

R^2^ = 0.609, R^2^adj = 0.602, F = 86.546, *P* < 0.01.

### 4.5 Mediation effect of artificial intelligence self-efficacy

PROCESS macro Model 4 was used to examine the mediating role of artificial intelligence self-efficacy. The results showed that artificial intelligence literacy was positively associated with artificial intelligence self-efficacy (b = 0.797, *P* < 0.01), and artificial intelligence self-efficacy was positively associated with innovative behavior (b = 0.404, *P* < 0.01). After artificial intelligence self-efficacy was included, the direct association between artificial intelligence literacy and innovative behavior remained statistically significant (b = 0.287, *P* < 0.01), as shown in Table 4. The bootstrap results showed that the direct effect of artificial intelligence literacy on innovative behavior was 0.287, and the mediating effect of artificial intelligence self-efficacy was 0.322. The 95% CIs for both the direct effect and the mediating effect did not include 0. This indicated that artificial intelligence self-efficacy partially mediated the relationship between artificial intelligence literacy and innovative behavior. The mediating effect accounted for 52.90% of the total effect, as shown in Table 5.

**Table 4:** Results of the mediation model.

Variables	AI self-efficacy			Nurse innovative behavior		
	b	t	95%CI	b	t	95%CI
AI literacy	0.797^**^	38.417	0.756, 0.838	0.287^**^	6.695	0.203, 0.372
AI self-efficacy	—			0.404^**^	9.412	0.320, 0.488
R^2^	0.635	—		0.431	—	
F	1475.849	—		320.404	—	

^**^P < 0.01.

**Table 5:** Total, direct, and indirect effects of artificial intelligence literacy on nurse innovative behavior.

Effect type	Effect	SE	LLCI	ULCI	Proportion of total effect (%)
Total effect	0.609	0.027	0.556	0.663	100%
Direct effect	0.287	0.043	0.203	0.372	47.10%
Indirect effect	0.322	0.041	0.243	0.403	52.90%

### 4.6 Moderating effect of organizational innovation climate and moderated mediation effect

PROCESS macro Model 14 was used to examine the moderating role of organizational innovation climate. The results showed that artificial intelligence literacy (b = 0.122, *P* < 0.01), artificial intelligence self-efficacy (b = 0.202, *P* < 0.01), and organizational innovation climate (b = 0.539, *P* < 0.01) were all positively associated with innovative behavior. The interaction term between artificial intelligence self-efficacy and organizational innovation climate was statistically significant (b = 0.051, *P* < 0.01), suggesting that the strength of the association between artificial intelligence self-efficacy and innovative behavior varied across levels of organizational innovation climate, as shown in Table 6 and Figure 2.

**Table 6:** Results of the moderated mediation model.

Variables	AI self-efficacy			Nurse innovative behavior		
	b	t	95%CI	b	t	95%CI
AI literacy	0.797^**^	38.417	0.756, 0.838	0.122^**^	3.231	0.048, 0.196
AI self-efficacy	—			0.202^**^	5.269	0.127, 0.277
Organizational innovation climate	—			0.539^**^	17.818	0.480, 0.599
AI self-efficacy × organizational innovation climate	—			0.051^**^	2.702	0.014, 0.088
R^2^	0.635	—		0.587	—	
F	1475.849	—		300.31	—	

^**^P < 0.01.

**Figure 2:**
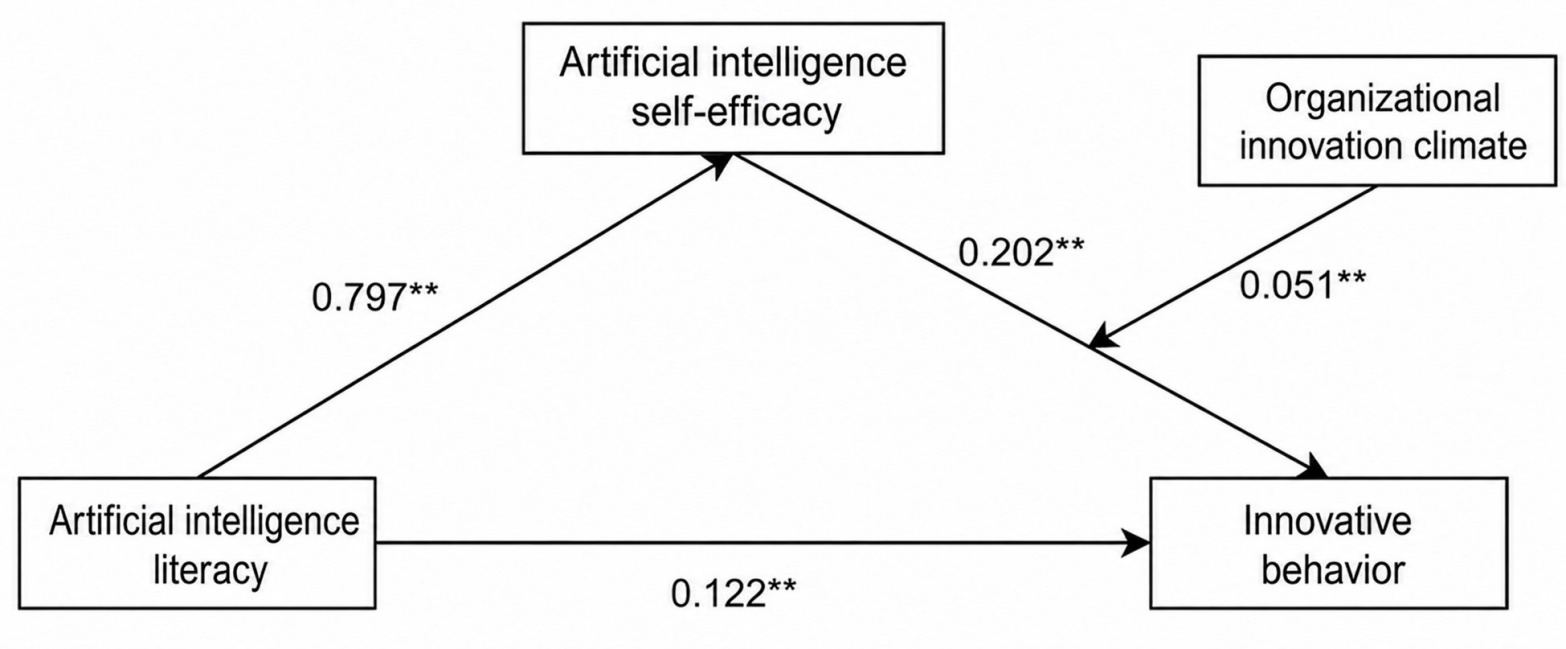
The final moderated mediation model. Standardized coefficients are presented. **P < 0.01.

Simple slope analysis showed that artificial intelligence self-efficacy was significantly and positively associated with innovative behavior at a low level of organizational innovation climate (b = 0.151, *P* < 0.01). At a high level of organizational innovation climate, the positive association between the two variables was stronger (b = 0.253, *P* < 0.01). These results suggested that the positive association between artificial intelligence self-efficacy and innovative behavior became stronger at higher levels of organizational innovation climate, as shown in Figure 3.

**Figure 3:**
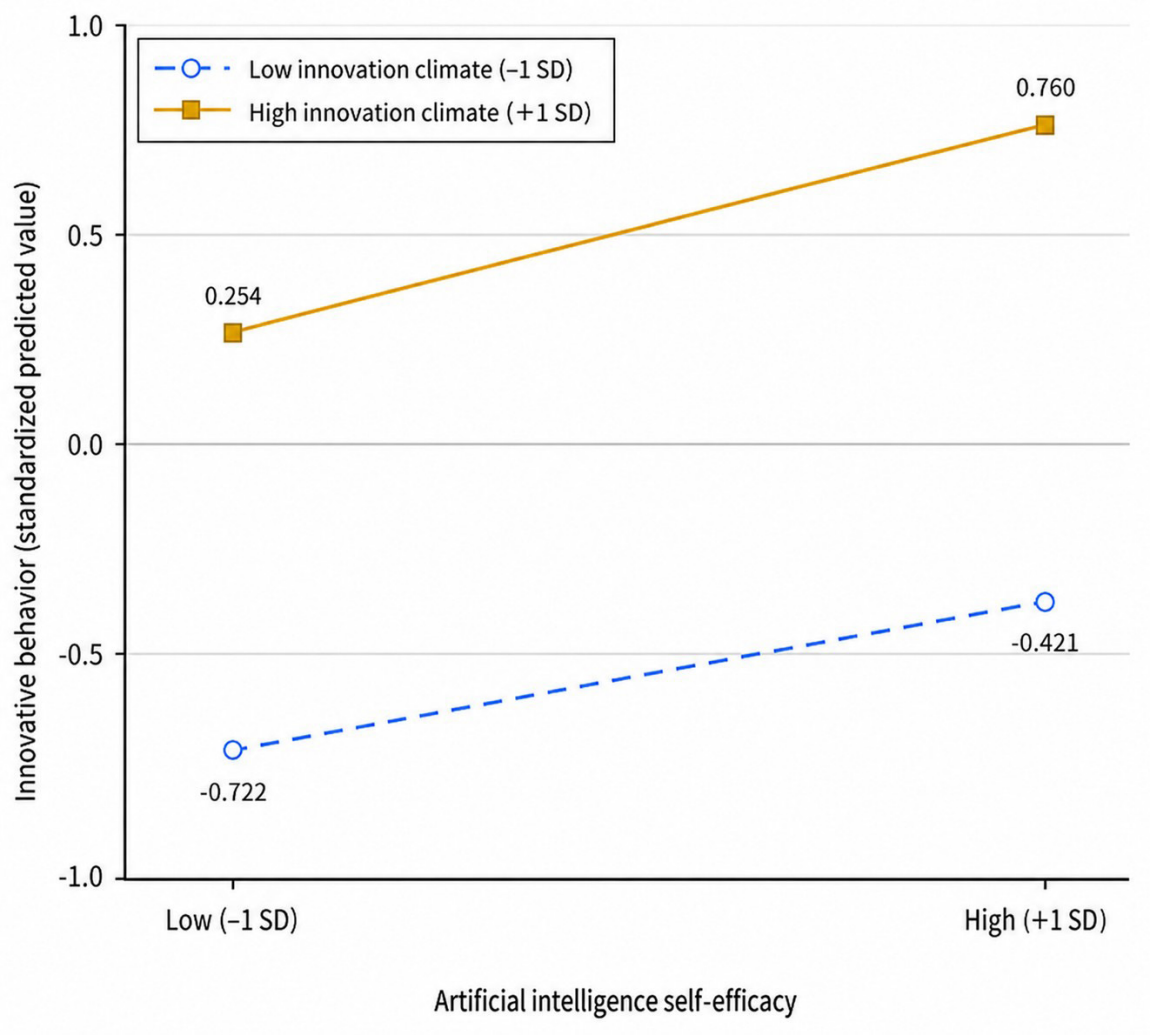
Organizational innovation climate as a moderator of the relationship between artificial intelligence self-efficacy and nurse innovative behavior.

Further analysis of the moderated mediation effect showed that the conditional indirect effects of the association between artificial intelligence literacy and innovative behavior through artificial intelligence self-efficacy were 0.120, 0.161, and 0.202 at low, mean, and high levels of organizational innovation climate, respectively. None of the bootstrap 95% CIs included 0. The index of moderated mediation was 0.041, and its bootstrap 95% CI did not include 0. These results suggested that the indirect effect varied across levels of organizational innovation climate and showed larger effect estimates at higher levels of organizational innovation climate, as shown in Table 7.

**Table 7:** Conditional indirect effects of artificial intelligence literacy on nurse innovative behavior through artificial intelligence self-efficacy at different levels of organizational innovation climate.

Level of organizational innovation climate	Effect	BootSE	BootLLCI	BootULCI
Low level (M−1 SD)	0.120	0.041	0.041	0.200
Mean level (M)	0.161	0.035	0.093	0.231
High level (M+1 SD)	0.202	0.038	0.130	0.277

## 5 Discussion

As artificial intelligence is gradually integrated into nursing practice, the relationship between artificial intelligence-related competencies and innovative behavior among clinical nurses has become an important issue in the development of intelligent nursing. Based on AMO theory, this study examined the factors associated with innovative behavior among clinical nurses and the patterns of their associations from the perspectives of ability, motivation, and opportunity. The results showed that educational level, professional title, and participation in research or innovation activities were associated with innovative behavior. Artificial intelligence literacy showed a positive association with innovative behavior. Artificial intelligence self-efficacy played a partial mediating role, and organizational innovation climate strengthened the positive relationship between artificial intelligence self-efficacy and innovative behavior. Notably, the strength of the associations between different factors and innovative behavior varied considerably, and the indirect effect through artificial intelligence self-efficacy and the moderating role of organizational innovation climate also differed in their practical implications.

### 5.1 Current status and related factors of innovative behavior among clinical nurses

The total score of innovative behavior among clinical nurses in this study was 37.93 ± 6.50, which was higher than the scores reported in previous studies, 32.66 ± 6.78 and 33.19 ± 6.71 (3, 27). This finding suggests that the participants in this study showed relatively active innovative behavior. This difference may be related to the sample composition. All nurses included in this study were from tertiary medical institutions. A relatively high proportion had bachelor's degrees and intermediate or senior professional titles, and some had experience in research or innovation improvement activities. These characteristics may have helped them identify clinical problems and propose and implement innovative solutions.

Multiple linear regression analysis showed that educational level, professional title, participation in research or innovation activities, artificial intelligence literacy, artificial intelligence self-efficacy, and organizational innovation climate were related factors of innovative behavior among clinical nurses. Higher educational levels and professional titles are usually associated with better knowledge integration, clinical experience, and resource coordination abilities. Participation in research or innovation activities also provides nurses with practical opportunities to identify problems and to test and promote innovative solutions (3, 28). However, organizational innovation climate showed the strongest association with innovative behavior (β = 0.502), exceeding those of artificial intelligence literacy (β = 0.124) and artificial intelligence self-efficacy (β = 0.211). This finding suggests that, compared with nurses' own ability and confidence in applying artificial intelligence, an organizational environment that encourages innovation and supports the implementation of new ideas is more closely associated with the actual expression of innovative behavior. Nurses' innovative behavior, from the generation of ideas to their promotion and implementation, involves not only individual ability but also corresponding organizational support and practical conditions (27). Therefore, when organizational support for innovation is limited, innovative ideas may still face barriers to implementation even when nurses possess adequate professional competence and a foundation for applying artificial intelligence. These findings further suggest that, beyond individual ability and motivation, the opportunity conditions provided by the organization may be particularly important to nurses' innovative behavior.

### 5.2 Relationship between artificial intelligence literacy and innovative behavior among clinical nurses: the mediating role of artificial intelligence self-efficacy

This study found that artificial intelligence literacy was positively correlated with innovative behavior among clinical nurses, and artificial intelligence self-efficacy partially mediated this relationship. These findings supported Hypothesis 1. The indirect effect through artificial intelligence self-efficacy accounted for 52.9% of the total effect, suggesting a relatively prominent indirect association between artificial intelligence literacy and innovative behavior. This finding indicates that the association between the two may be related not only to nurses' artificial intelligence knowledge and abilities but also to their confidence in applying artificial intelligence. Previous studies provide some support for this finding. Artificial intelligence self-efficacy has been reported to mediate the relationship between artificial intelligence literacy and intention to use artificial intelligence (10, 29) and to be closely associated with nurses' innovative behavior (30, 31). Building on these findings, the present study further focused on innovative behavior among clinical nurses, suggesting that artificial intelligence self-efficacy also warrants attention in the association between artificial intelligence literacy and nurse innovative behavior. Previous studies mainly focused on nursing students or nurses from a single specialty. In contrast, this study provided further evidence on innovative behavior among clinical nurses from different types of tertiary medical institutions.

Artificial intelligence literacy helps nurses understand the functions and limitations of technology, assess appropriate application scenarios, and pay attention to information quality and potential risks (32, 33). Related studies have shown that clear system logic and decision-making evidence can enhance nurses' trust in technology and their sense of control. Greater familiarity with artificial intelligence is also associated with more positive perceptions of professional value and a stronger intention to use it (34, 35). Such knowledge and experience may reduce nurses' uncertainty when facing new technologies and strengthen their confidence in using artificial intelligence to solve clinical problems. Innovative behavior involves the generation, promotion, and implementation of new methods or solutions. This process is often accompanied by additional effort, technical difficulties, and uncertainty about outcomes. Stronger artificial intelligence self-efficacy may help nurses maintain confidence and take action during this process. It may also encourage them to use artificial intelligence to continuously advance innovation. Therefore, artificial intelligence self-efficacy may be an important psychological factor linking artificial intelligence literacy to innovative behavior among clinical nurses. From the perspective of AMO theory, these findings suggest that individual ability and motivation are not independent of each other, and that artificial intelligence-related abilities and confidence in their application may both be associated with nurses' innovative behavior.

### 5.3 Moderating role of organizational innovation climate

This study found that organizational innovation climate positively moderated the relationship between artificial intelligence self-efficacy and nurses' innovative behavior. The conditional indirect effect also became stronger as the level of organizational innovation climate increased. These findings supported Hypothesis 2. The results showed that artificial intelligence self-efficacy was positively associated with innovative behavior across different levels of organizational innovation climate, and both the association and the conditional indirect effect were stronger at higher levels of organizational innovation climate. This suggests that the moderating role of organizational innovation climate was mainly reflected in the closer positive association between artificial intelligence self-efficacy and nurse innovative behavior at higher levels of organizational innovation climate. Previous studies have shown that organizational innovation climate may be related to nurses' innovative behavior through self-efficacy (18) and may strengthen the positive relationship between self-efficacy and innovative behavior (19). Research in the context of artificial intelligence has also found that higher organizational artificial intelligence readiness can reduce the negative effect of perceived technological substitution on nurses' innovative behavior (36). Compared with previous studies that mainly focused on the direct association between the organizational environment and innovative behavior, the present study further suggests that organizational innovation climate may also be related to differences in the strength of the association between individuals' confidence in applying artificial intelligence and innovative behavior, further highlighting the importance of organizational context in artificial intelligence-related nursing innovation.

Artificial intelligence self-efficacy can strengthen nurses' confidence in using intelligent technologies to solve clinical problems. However, using artificial intelligence to support the improvement of nursing problems and innovation still requires corresponding organizational support. A positive organizational innovation climate can reduce uncertainty in the application of artificial intelligence technologies and the implementation of innovation through recognition of innovation, resources, and management support (6, 37). In the context of artificial intelligence, sufficient time, technical support, and clear implementation procedures may help nurses improve innovative solutions and control potential risks (38). Organizational innovation climate may also be related to innovative behavior through knowledge sharing (20). Open communication can promote the integration of clinical experience and artificial intelligence-related information and help nurses improve innovative solutions. In contrast, when resources are insufficient, procedures are unclear, or collaborative support is limited, nurses may reduce their innovative attempts because of concerns about implementation, even if they have strong artificial intelligence self-efficacy. According to AMO theory, artificial intelligence self-efficacy provides the motivation for nurses to engage in innovative behavior, while organizational innovation climate provides the necessary practical opportunities. Both are associated with the innovative behavior of clinical nurses in the context of artificial intelligence. The present findings further suggest that opportunity factors may not only be associated with innovative behavior together with ability and motivation, but may also serve as important contextual conditions related to the strength of the association between motivation-related psychological beliefs and innovative behavior. These findings provide additional evidence for understanding the contextual role of opportunity within AMO theory.

### 5.4 Theoretical significance and implications for nursing management

Based on AMO theory (39), this study further clarifies the external contextual role of opportunity in the relationship between motivation and innovative behavior. The results showed that the positive association between artificial intelligence self-efficacy and nurse innovative behavior was stronger at higher levels of organizational innovation climate. This finding suggests that opportunity factors may not only be directly associated with innovative behavior, but may also represent an important condition related to the strength of the association between motivation-related beliefs and innovative behavior. Organizational context may therefore help explain differences in the strength of this association. In this respect, the present study further extends understanding of the conditions under which opportunity operates within AMO theory, with particular attention to contextual differences in the relationship between motivation and behavior, and provides additional empirical evidence for the application of AMO theory in the field of artificial intelligence in nursing.

In nursing management, artificial intelligence training that combines knowledge acquisition with context-based practice may be explored according to clinical needs, while attention should also be paid to nurses' feedback and changes in confidence during learning and application. Nursing managers may also consider strengthening organizational support through work scheduling, technical resources, and interprofessional collaboration. Under the premise of complying with standards for artificial intelligence use and ensuring safety, appropriate opportunities for nursing innovation may be provided in clinical practice.

## 6 Strengths and limitations

### 6.1 Strengths

This study included 850 clinical nurses from six tertiary medical institutions in Yueyang City. The institutions included both general hospitals and specialized hospitals. The sample size was relatively large, and the sample sources showed a certain degree of diversity. This study included artificial intelligence self-efficacy as a distinctive variable. Compared with general self-efficacy, it can better reflect nurses' confidence in learning, adapting to, and using artificial intelligence. In addition, this study simultaneously examined the mediation effect, moderation effect, and moderated mediation effect. It therefore provided a relatively systematic examination of the relationships among artificial intelligence literacy, confidence in applying artificial intelligence, organizational environment, and innovative behavior among clinical nurses.

### 6.2 Limitations and future research

This study used a cross-sectional design, and all variables were measured at the same time point. Therefore, their temporal sequence and causal relationships could not be determined. Future studies may use longitudinal, multi-wave, or intervention designs to further examine the dynamic relationships among these variables. Second, although Harman's single-factor test did not identify obvious common method bias, all data were collected using self-report questionnaires. Therefore, self-report bias may still exist. Future studies may combine evaluations from head nurses or colleagues and include objective indicators, such as nursing innovation projects, quality improvement outcomes, patents, and records of artificial intelligence tool use, to improve the reliability of the findings. Finally, the sample was limited to tertiary medical institutions in Yueyang City. Potential clustering of nurses within hospitals was also not further considered. Future studies may include multiple regions, different hospital levels, and multiple centers, and retain anonymized hospital identifiers while protecting privacy to enable the assessment and analytical handling of within-hospital correlations.

## 7 Conclusion

This study suggests that innovative behavior among clinical nurses is closely associated not only with their ability to use artificial intelligence, but also with their confidence in applying artificial intelligence and organizational support for innovation. Artificial intelligence literacy, artificial intelligence self-efficacy, and organizational innovation climate were all positively associated with nurse innovative behavior. Artificial intelligence self-efficacy showed an indirect mediating association between artificial intelligence literacy and innovative behavior, while organizational innovation climate was related to variation in the strength of the association between artificial intelligence self-efficacy and innovative behavior. Nursing managers may consider nurses' artificial intelligence competencies, confidence in applying artificial intelligence, and the organizational innovation environment, with artificial intelligence competency development, practice in clinical contexts, and organizational support as potential management targets for promoting nurse innovative behavior. The effectiveness of these approaches should be further evaluated in future intervention studies.

## Data Availability

The raw data supporting the conclusions of this article will be made available by the authors, without undue reservation.
